# A Non-Destructive Health Evaluation Method for Wooden Utility Poles with Frequency-Modulated Empirical Mode Decomposition and Laplace Wavelet Correlation Filtering

**DOI:** 10.3390/s22114007

**Published:** 2022-05-25

**Authors:** Xiaoli Zhang, Ji Yang, Weidong Zhu, Gang Li

**Affiliations:** 1 Key Laboratory of Road Construction Technology and Equipment, Ministry of Education, Chang’an University, Xi’an 710064, China; lilyzhang@chd.edu.cn (X.Z.); toddyang1994@163.com (J.Y.); 2 Department of Mechanical Engineering, University of Maryland, Baltimore County, Baltimore, MD 21250, USA; gangli@umbc.edu

**Keywords:** non-destructive health evaluation, wooden utility pole, FM-EMD, Laplace wavelet correlation filtering, instantaneous frequency, damping factor

## Abstract

Wooden utility poles are one of the most commonly used utility carriers in North America. Even though they are given different protection treatments, wooden utility poles are prone to have defects that are mainly caused by temperature, oxygen, moisture, and high potential hydrogen levels after decades of being exposed in open-air areas. In order to meet the growing demand regarding their maintenance and replacement, an effective health evaluation technology for wooden utility poles is essential to ensure normal power supply and safety. However, the commonly used hole-drilling inspection method always causes extra damage to wooden utility poles and the precision of health evaluation highly relies on technician experience at present. Therefore, a non-destructive health evaluation method with frequency-modulated empirical mode decomposition (FM-EMD) and Laplace wavelet correlation filtering based on dynamic responses of wooden utility poles was proposed in this work. Specifically, FM-EMD was used to separate multiple confusing closely-spaced vibration modes due to nonlinear properties of wooden utility poles into several single modes. The instantaneous frequency and damping factor of the decomposed signal of each single mode of the dynamic response of a wooden utility pole could be determined using Laplace wavelet correlation filtering with high precision. The health status of a wooden utility pole could then be estimated according to the extracted instantaneous frequency and damping factor of the decomposed signal of each single mode. The proposed non-destructive health evaluation method for wooden utility poles was tested in the field and achieved successful results.

## 1. Introduction

Wooden utility poles are widely used to support overhead power transmission lines and various other public utilities all over the world, such as electric lines, fiber optic cables, and related equipment such as transformers and streetlights. Their widespread usage is due to the advantages of wooden material attributes, which can save on costs and provide enough strength and great flexibility to place cable hardware. It can be easily acquired from nature with less environmental pollution than other options.

**Figure 1:**
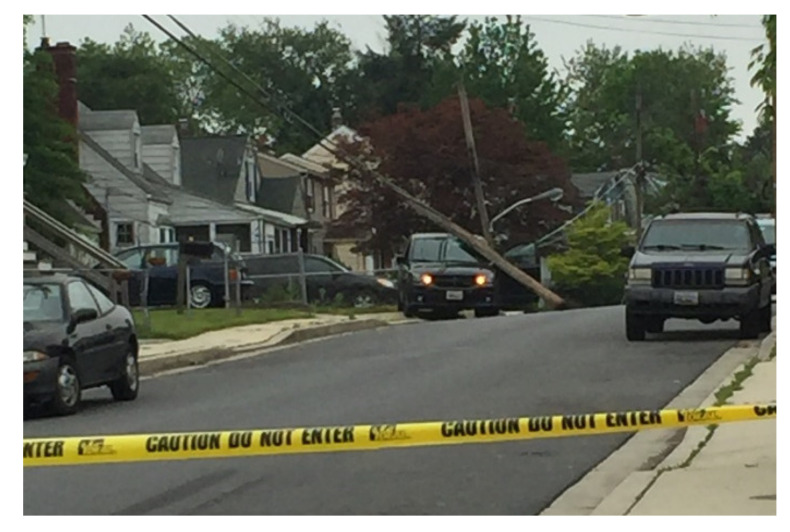
Accident of a wooden utility pole failure in Baltimore in 2019.

**Figure 2:**
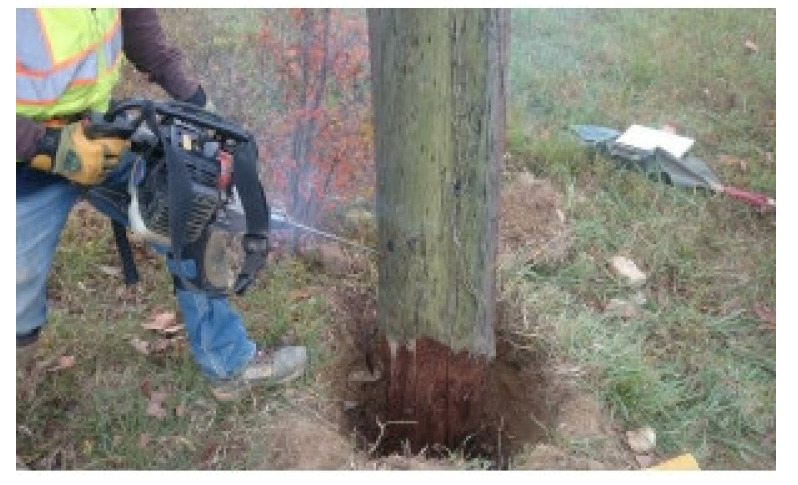
Conventional wooden utility pole inspection method.

Wooden utility poles were first used with telegraph systems in the U.S. in the mid-19th century to build a line between Baltimore and Washington, D.C. According to the survey by Baltimore Gas and Electric Company (BGE), wooden utility poles are widely used in North America and southern yellow pine is a major wood type used by BGE. However, wooden utility poles are facing more and more severe erosion threats after decades of exposure to the weather, even though these poles are under anti-decay protection [1]. According to statistics, over 38,500 wooden utility poles required reliability inspection in 2016. Wooden utility pole failure can cause power interruption, damage to nearby buildings and vehicles, and accidents that can affect human life. Figure 1 shows a scene of a wooden utility pole failure in Baltimore on 9 May 2019. All wooden utility poles in the U.S. are regularly examined via hole-drilling and sample extraction at their bases to check for conditions of decay of the poles, as shown in Figure 2. Generally, there are two randomly drilled holes at different heights, from heights close to the ground and close to a man’s height. Technicians insert a steel bar into a sampling hole to judge the remaining radius of wood, and the condition of surface decay is estimated via visual examination. However, the commonly used hole-drilling inspection method has at least the following drawbacks. The inspection accuracy is unreliable to some extent since it mainly depends on the drilling position as well as technician experience. More importantly, it does damage to the pole structure and affects the remaining life of the pole. This is the reason why a non-destructive health evaluation method is imperative for wooden utility poles.

There are several types of non-destructive damage detection and health evaluation methods for wooden utility poles. Wu et al. [2] proposed a conventional measurement method for wood component moisture content through channel state information of Wi-Fi signals and their corresponding high-precision hybrid feature extraction via a bimodal deep extreme learning machine. An ultrasonic tomography inspection method for wooden utility poles was developed by Tomikawa et al. [3], the idea of which was based on the conventional X-ray technology that measures data on each section layer, and the image of each section layer shows the rotten wood area and healthy wood area with particular simplicity. By gathering multiple section layers, an entire X-ray image for a wooden utility pole can be captured. However, the drawback of the ultrasonic inspection method is that ultrasound does not linearly propagate because the Young’s modulus of the wooden utility pole varies between the sap and center. Moreover, ultrasound cannot penetrate a rotten wood area. If ultrasound meets a pocket, no further detection can be reached to the core of a wooden utility pole; thus, it cannot have a complete measurement of a certain section. Krause et al. [4] presented two non-destructive testing methods based on elastic wave propagation and reflection for condition assessment of structural timber. The first approach uses ultrasonic echoes for testing wooden building elements. The second approach uses guided waves for testing wooden pole and pile structures. Li et al. [5] investigated wave propagation in wooden utility poles in combination with wavelet transform analysis for identification of conditions and underground depths of embedded wooden utility poles in service. Wyckhuyse and Maldague [6,7] adopted infrared thermography to inspect wooden utility poles. The infrared thermography technology built a model based on different moisture contents to compare wood thermal properties. However, a signal of infrared thermography would be attenuated by exterior wood. One cannot detect any interior decay of a pole unless the decaying position is close to the surface of the wooden utility pole.

Similar to the methods mentioned above, Tsang et al. [8] used the acoustic impulse response to analyze conditions of different wooden utility poles and conducted sound energy analysis by combining fuzzy logic and frequency analysis methods. Xu et al. [9] used an acoustic separation and enhancement algorithm to explore the use of acoustic impact tests to evaluate the conditions of hardwood logs regarding their internal decay, cracks, and void. Raczkowski et al. [10] achieved detection of the early stage of wood decay based on the acoustic emission method. However, the acoustic emission method is sensitive to material properties and prone to be affected by noise. In order to meet practical engineering concerns for inspection convenience and accuracy, a non-destructive health evaluation method of wooden utility poles with their instantaneous frequencies and damping factors extracted from their dynamic response signals was proposed in this work.

Modal parameters of a structure usually play an important role in structural health evaluation since damage often affects its dynamic behaviors and triggers changes in its dynamic characteristics, such as natural frequencies, damping ratios, and mode shapes [11,12]. Due to the relationship between modal parameters and structural health conditions, modal parameters of a monitored structure are useful for non-destructive health evaluation. However, a directly acquired dynamic signal of a nonlinear structure usually shows confusing multimodal characteristics and each single mode can contain its own modal information. Therefore, it is necessary to decompose a confusing multimodal signal into several single-mode signals using the frequency-modulated empirical mode decomposition (FM-EMD) method. Commonly, modal parameters hidden in each single mode are extracted using the Hilbert transform to draw the logarithmic amplitude–frequency diagram and phase–frequency diagram [13,14]. Lei and Zuo [15] proposed a fault diagnosis method for rotating machinery based on ensemble empirical mode decomposition to solve its mode-mixing problem when decomposing multiple-order modes and obtain its modal parameters using an improved Hilbert–Huang transform (HHT). Das et al. [16] presented a vibration-based damage detection method for wooden utility poles based on the wavelet packet transform (WPT) and an improved HHT. The logarithmic amplitude–frequency and phase–frequency relationships could become linear using curve fitting. Modal parameters of a single mode could then be obtained via calculating the slopes of curve-fitted lines. Inevitably, there are fitting errors for the logarithmic amplitude–frequency and phase–frequency lines obtained via curve fitting, which affects the accuracy of modal parameter identification. In order to improve the accuracy of modal parameter identification, correlation filtering based on the Laplace wavelet was introduced [17]. Some studies [18,19,20] presented some case studies of Laplace wavelet correlation filtering methods for identifying natural frequencies and damping ratios of different structures. Since the correlation filtering technology is based on the principle of waveform similarity matching and the waveform of an impact response signal is often similar to the Laplace wavelet, the natural frequency and damping ratio of each single mode can be picked up using Laplace wavelet correlation filtering with high precision. Zhang et al. [21] proposed an inverse decaying FM-EMD method combined with Laplace wavelet correlation filtering for identifying bolt tightness in an aero-engine rotor assembly by extracting its natural frequency information. However, damping factors of the aero-engine rotor assembly were not studied.

Traditional EMD can separate multiple modes when frequency ratios are larger than 2. If the frequency ratio of two adjacent modes is less than 2, the conditions of neighboring intrinsic mode functions (IMFs) cannot be satisfied, which causes the inability of EMD to separate closely spaced modes. The current improved EMD mainly focuses on frequency analysis. However, damping factors, which are important factors of closely spaced modes, are usually neglected. Moreover, instantaneous frequencies and damping factors in decomposed single modes are always obtained based on the HHT to plot logarithmic amplitude–frequency and phase–frequency diagrams, and to retain their linear relationships using curve fitting, whose slopes can be calculated from these lines. However, the logarithmic amplitude–frequency and phase–frequency lines obtained using curve fitting differ from the original data, which affects the accuracy of structural health evaluation.

The main objective of this study was to propose a non-destructive health evaluation method with FM-EMD and Laplace wavelet correlation filtering for health evaluation of wooden utility poles. The proposed FM-EMD method could separate closely spaced modes into multiple single modes by shifting a frequency to transform closely spaced modes from a high-frequency zone to a low-frequency zone. Subsequently, a reverse transformation was performed to ensure the accuracy of the instantaneous frequency and damping factor of the decomposed signal of each single mode. Laplace wavelet correlation filtering was used to pick up the instantaneous frequency and damping factor of the decomposed signal of each single mode. Both instantaneous frequencies and damping factors of wooden utility poles were studied here, which are crucial for health evaluation of wooden utility poles.

The rest of this paper is organized as follows. Theories of FM-EMD and Laplace wavelet correlation filtering are briefly reviewed in Section 2. Section 3 explains the proposed method with FM-EMD and Laplace wavelet correlation filtering to extract instantaneous frequencies and damping factors of decomposed signals of dynamic responses of a structure from confusing multimodal signals. Section 4 explores the proposed method for health evaluation of wooden utility poles in the field. Finally, some conclusions are given in Section 5.

## 2. Theoretical Background

### 2.1. FM-EMD Method

FM-EMD is an improvement over the traditional EMD regarding solving the multiple-mode confusion problem, which is based on the frequency shifting process. It can overcome the drawbacks of traditional EMD regarding the failure to separate multiple confusing modes. Specifically, given a measured signal *x*(*t*), where *t* is time, that has multiple modes and many spectral lines in the frequency spectrum, FM-EMD performs frequency modulation by shifting the frequency from a higher frequency zone to a lower frequency zone and adjusting the amplitude ratio to reduce the mixing degree of multiple modes before the FM-EMD decomposing process. In order to improve the efficiency of signal processing, a measured real signal *x*(*t*) is first transformed to a complex signal *X*(*t*) using the Hilbert transform that can be represented as

(1) $$H\left[x\left(t\right)\right]=\frac{1}{\pi }P\int_{-\infty }^{\infty }\frac{x\left(t\prime \right)}{t-t\prime }dt^{\prime }=x\left(t\right)\cdot \frac{1}{\pi t},$$

(2) $$X\left(t\right)=x\left(t\right)+\tilde{i}H\left[x\left(t\right)\right],$$

where $\tilde{i}$=$\sqrt{-1}$ and *P* is the principal value of the singular integral. An appropriate modulation frequency $\omega_{0}$ is selected to transform the analytical signal *X*(*t*) from a higher frequency zone to an analytical signal *Z*(*t*) in a lower frequency zone. The analytical signal *Z*(*t*) can be represented as

(3) $$Z\left(t\right)=X\left(t\right)\cdot e^{-\tilde{i}\cdot \omega_{0}\cdot t}=Z_{r}\left(t\right)+\tilde{i}Z_{j}\left(t\right),$$

where $\omega_{0}$ is a modulation frequency that can be set based on actual demands, and *Z_r_*(*t*) and *Z_j_*(*t*) are real and imaginary parts of *Z*(*t*), respectively. Assume that there are two closely spaced frequencies *f*_1_ and *f*_2_. FM-EMD can effectively separate multiple closely spaced spectral lines if the selected $\omega_{0}$ can make the frequency ratio and amplitude ratio of two frequencies satisfy the requirements [22]

(4) $$\left\{\begin{array}{c} f_{1}/f_{2}<0.5\\ a_{1}f_{1}>a_{2}f_{2} \end{array}\;\;\right.,$$

where *f*_1_ < *f*_2_, and *a*_1_ and *a*_2_ are the amplitudes of *f*_1_ and *f*_2_, respectively. Therefore, the mixing degree of multiple modes is decreased by a shifting frequency, which can be successfully decomposed using FM-EMD. All the local maxima and minima of *Z_r_*(*t*) and *Z_j_*(*t*) are separately identified, and their upper and lower envelopes are then formed by interpolating the local maxima and minima of *Z_r_*(*t*) and *Z_j_*(*t*) with cubic spline lines, respectively. All the extrema of *Z_r_*(*t*) and Z*_j_*(*t*), which are larger than the local maxima of *Z_r_*(*t*) and *Z_j_*(*t*) or smaller than their local maxima, should be covered in these two envelopes. The mean values of the upper and lower envelopes of *Z_r_*(*t*) and *Z_j_*(*t*) are denoted as *med_r_*_1_ and *med_j_*_1_, respectively. The differences between *Z_r_*(*t*) and its mean value *med_r_*_1_ and between *Z_j_*(*t*) and its mean *med_j_*_1_ are

(5) $$\left\{\begin{array}{c} h_{r1}=Z_{r}\left(t\right)-med_{r1}\\ h_{j1}=Z_{j}\left(t\right)-med_{j1} \end{array}\right..$$

The EMD method was developed to decompose a signal into IMFs based on the assumption that a signal consists of different simple IMFs, which is some collection of individual and almost mono-component signals. An IMF is a function that satisfies the following two conditions: (1) the number of extrema and the number of zero-crossings must either be the same or differ at most by one in the whole data set, and (2) the mean value at any point between envelopes defined by local maxima and minima is zero [23]:

(a) The number of extremal points *N_e_*, including the maxima and minima, and the number of zero-crossing points *Nz* must satisfy
  (6) $$\left(N_{z}-1\right)\leq N_{e}\leq \left(N_{z}+1\right).$$
(b) At any point, the local maxima *f_max_*(*t*) and local minima *f_min_*(*t*) must satisfy
  (7) $$\frac{f_{max}\left(t\right)+f_{min}\left(t\right)}{2}=0.$$

The first components of the IMFs of *Z_r_*(*t*) and *Z_j_*(*t*) are denoted as *c_r_*_1_ and *c_j_*_1_, respectively. By subtracting *c_r_*_1_ and *c_j_*_1_ from their original data, residues *r_r_*_1_ and *r_j_*_1_ of *Z_r_*(*t*) and *Z_j_*(*t*) can be represented as

(8) $$\left\{\begin{array}{c} r_{r1}=Z_{r}-c_{r1}\\ r_{j1}=Z_{j}-c_{j1} \end{array}\right.,$$

respectively. If *r_r_*_1_ and *r_j_*_1_ still contain information about other components of the IMFs of *Z_r_*(*t*) and *Z_j_*(*t*), set *r_r_*_1_ and *r_j_*_1_ as the new data and repeat the above sifting process to obtain the first components *c_r_*_1_ and *c_j_*_1_ of the IMFs of *Z_r_*(*t*) and *Z_j_*(*t*), respectively. This sifting process must be repeated until *h_r_* and *h_j_* satisfy

(9) $$\left\{\begin{array}{c} S_{rd}=\int_{0}^{T}\frac{{\left|\left(h_{r\left(k-1\right)}\left(t\right)-h_{rk}\left(t\right)\right)\right|}^{2}}{h_{rk}^{2}\left(t\right)}dt\\ S_{jd}=\int_{0}^{T}\frac{{\left|\left(h_{j\left(k-1\right)}\left(t\right)-h_{jk}\left(t\right)\right)\right|}^{2}}{h_{jk}^{2}\left(t\right)}dt \end{array}\right.,$$

where *k* is the number of orders of the IMFs of *Z_r_*(*t*) and *Z_j_*(*t*), *h_r_*_(*k*−1)_(*t*) and *h_r_*_(*k*)_(*t*) are the time series of two consecutive processing results in the sifting IMFs of *Z_r_*, *h_j_*_(*k*−1)_(*t*) and *h_j_*_(*k*)_(*t*) are the time series of two consecutive processing results in the sifting IMFs of *Z_j_*, and *T* is the time span of the signal. When *S_rd_* and *S_jd_* reach values in the range of 0.2~0.3, based on the practical experience [24], the sifting process stops. Finally, *Z_r_*(*t*) and *Z_j_*(*t*) are separately decomposed into *n* IMFs, which are

(10) $$\left\{\begin{array}{c} Z_{r}\left(t\right)=\overset{n}{\underset{i=1}{\sum }}c_{ri}+r_{rn}\\ Z_{j}\left(t\right)=\overset{n}{\underset{i=1}{\sum }}c_{ji}+r_{jn} \end{array}\right.,$$

where *i* is the mode number. All IMFs are the results of decomposed data *Z*(*t*) that differ from the original signal *x*(*t*). Due to the inverse decay and frequency shifting, the instantaneous frequency and damping factor of *Z*(*t*) decrease. The decomposed IMFs of *Z*(*t*) are

(11) $$\left\{\begin{array}{c} X_{r}\left(t\right)=\overset{n}{\underset{i=1}{\sum }}c_{ri}\cdot e^{\tilde{i}\cdot \omega_{0}\cdot t}+r_{rn}\cdot e^{\tilde{i}\cdot \omega_{0}\cdot t}\\ X_{j}\left(t\right)=\overset{n}{\underset{i=1}{\sum }}c_{ji}\cdot e^{\tilde{i}\cdot \omega_{0}\cdot t}+r_{jn}\cdot e^{\tilde{i}\cdot \omega_{0}\cdot t} \end{array}\right.,$$

where *C_i_* are the IMFs that are separated using FM-EMD, and each IMF is a single-mode signal. Based on Equation (11), the original signal *x*(*t*) can be written as

(12) $$x\left(t\right)=Re\left(X\left(t\right)\right)=Re\left(X_{r}\left(t\right)+\tilde{i}\cdot X_{j}\left(t\right)\right)=\sum_{i=1}^{n}C_{i}+R_{n}.$$

### 2.2. Laplace Wavelet Correlation Filtering

#### 2.2.1. Laplace Wavelet

A Laplace wavelet is a unilateral attenuation function, which can be defined as [17]

(13) $$\psi \left(\omega ,\zeta ,\tau ,t\right)=\psi_{r}\left(t\right)=\left\{\begin{array}{cc} Ae^{-\frac{\zeta }{\sqrt{1-\zeta^{2}}}\omega \left(t-\tau \right)}e^{-j\omega \left(t-\tau \right)}, & t\epsilon \left[\tau ,\tau +W_{s}\right],\\ 0, & \mathrm{elsewise} \end{array}\right.$$

where *ω*∈R^+^ is the wavelet center frequency in the frequency domain; *ζ*∈[0, 1) ⊂R^+^ is the damping factor that controls the decay rate of the exponential envelope in the time domain and hence regulates the resolution of the wavelet, which simultaneously corresponds to the frequency bandwidth of the wavelet in the frequency domain; *τ*∈R^+^ is the time index; *A* is an arbitrary scaling factor; and *W_s_* is the range that ensures the Laplace wavelet to be compactly supported, which has a nonzero finite length.

The Laplace wavelet is constructed in consideration of the engineering application demand. It has properties of unilateral attenuation in the absence of orthogonality properties, which means that the Laplace wavelet cannot be used by the traditional wavelet transform. Matching pursuit is a self-adaptive wavelet decomposition method that can decompose a signal into a linear expansion of a group of basic functions [25]. These basic functions are all from the wavelet dictionary and can best match the signal structure. Correlation filtering is, in essence, a matching pursuit process.

#### 2.2.2. Correlation Filtering

The correlation between a dynamic signal of a system *x*(*t*) and its Laplace wavelet can be obtained using an inner product operation that can be represented as

(14) $$\langle \psi_{r}\left(t\right),x\left(t\right)\rangle =||\psi_{r}||{}_{2}||x||{}_{2}||\cos\theta ,$$

where *ψ_r_*(*t*) is one of the dictionary wavelets *Ψ* that are generated by discrete gridding of the parameter space, which are

(15) $$\left\{\begin{array}{c} \Omega =\left\{\omega_{1},\omega_{2}\ldots \omega_{p}\right\}\subset R^{+}\\ Z=\left\{\zeta_{1},\zeta_{2}\ldots \zeta_{q}\right\}\subset \left[0,\left.1\right)\right.\\ T=\left\{\tau_{1},\tau_{2}\ldots \tau_{r}\right\}\subset R\\ \gamma \in \Gamma =\Omega \times Z\times T\\ \Psi =\left\{\psi \left(\omega ,\zeta ,\tau ,t\right):\omega \in \Omega ,\zeta \in Z,\tau \in T\right\} \end{array}\right..$$

The similarity between the signal *x*(*t*) generated from the system and the properties of its Laplace wavelet can be represented as

(16) $$\;K_{r}=\cos\theta =\sqrt{2}\frac{\left|\langle \psi_{r}\left(t\right),x\left(t\right)\rangle \right|}{||\psi_{r}||{}_{2}||x||{}_{2}},$$

where *K_r_* is a correlation coefficient that is determined by the parameter vector {*ω*, *ζ*, *τ*}. For a given time index *τ*, the inner product operation in Equation (14) searches for a maximum correlation coefficient across the instantaneous frequency *ω* and damping factor *ζ* of a decomposed signal. Define $\bar{\omega }$ and $\bar{\zeta }$ as a given frequency and damping factor of peak correlation in the Laplace wavelet. The relationship between the maximum correlation coefficient *K_τ_* and a local peak value $K_{r}^{\tau }$ is

(17) $$K_{\tau }=\underset{\begin{array}{c} \omega \in \Omega \\ \zeta \epsilon Z \end{array}}{\max}K_{r}^{\tau }=K_{\left\{\bar{\omega },\bar{\zeta ,}\tau \right\}}.$$

The correlation filter approach can calculate the similarity between *x*(*t*) and its Laplace wavelet, which can also work as a transform from the time domain to a parameter domain. The given frequency $\bar{\omega }$ and damping factor $\bar{\zeta }$ associated with the maximum correlation coefficient *K_τ_* are based on the measured data *x*(*t*).

## 3. Proposed Method with FM-EMD and Laplace Wavelet Correlation Filtering for Non-Destructive Health Evaluation

**Figure 3:**
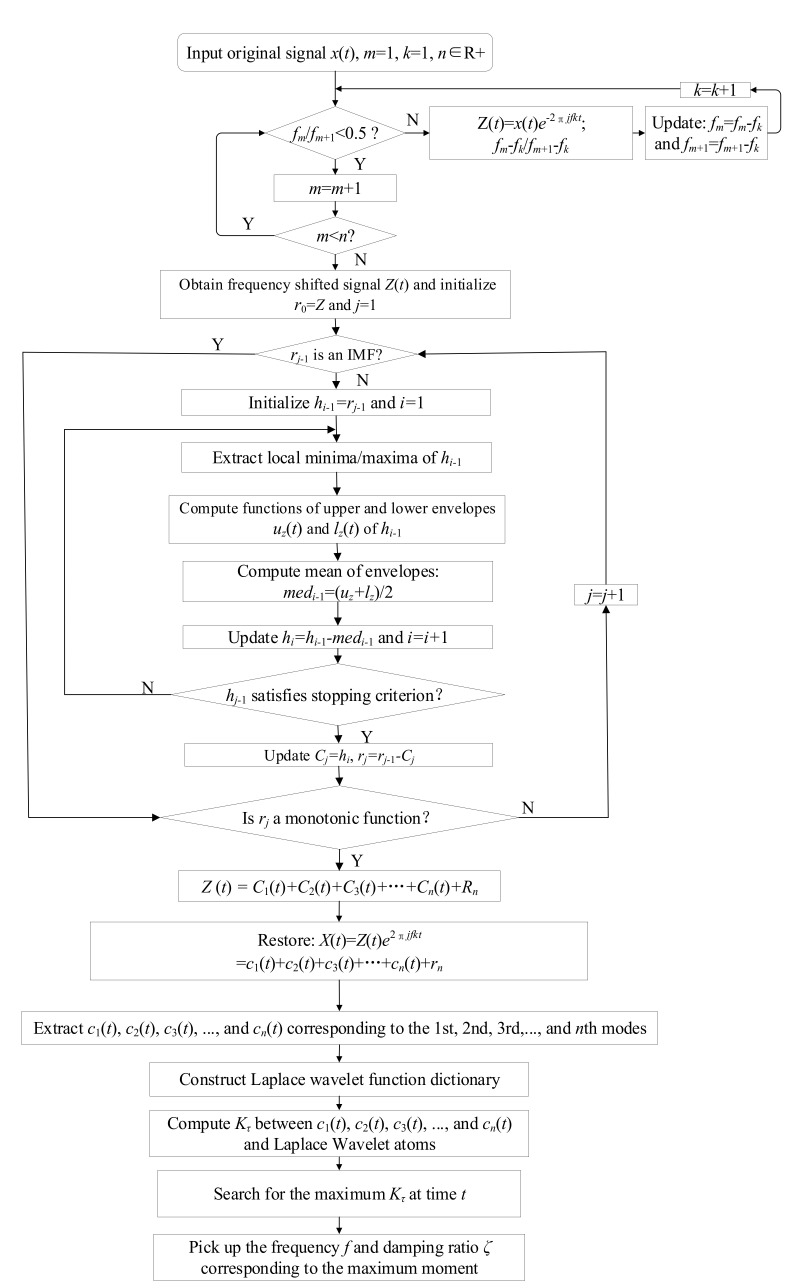
Flow chart of the proposed method for non-destructive health evaluation.

Since the correlation filtering technology is based on the principle of the waveform similarity matching theory and the waveform of an impact response signal often behaves similar to the Laplace wavelet, the instantaneous frequency and damping factor of the decomposed signal of each single mode can be obtained using Laplace wavelet correlation filtering with high precision. Therefore, a non-destructive health evaluation method with FM-EMD and Laplace wavelet correlation filtering was proposed for health evaluation of wooden utility poles. In order to clearly describe the proposed non-destructive health evaluation method with FM-EMD and Laplace wavelet correlation filtering, a flow chart of the proposed method is shown in Figure 3.

Step 1: Initialize parameters and variables of the proposed method. Define the number of modes as *n* and the real signal *x*(*t*) collected from accelerometers installed on tested wooden poles is then transformed to a complex signal with the Hilbert transform to obtain the analytical signal *X*(*t*) using Equations (1) and (2). Choose an appropriate modulation frequency $\omega_{0}$ to transform the analytical signal from a high-frequency zone to a low-frequency zone using Equation (3).

Step 2: Only if the selected $\omega_{0}$ can make the frequency ratio and amplitude ratio of two closely spaced spectral lines at frequencies *f*_1_ and *f*_2_ satisfy the requirements in Equation (4), the proposed FM-EMD method can be performed with the following steps 2.1–2.6.

Step 2.1: Identify all extrema of *Z_r_*(*t*) and *Z_j_*(*t*) and collect all local maxima of *Z_r_*(*t*) and *Z_j_*(*t*) using cubic spline lines as upper envelopes of *Z_r_*(*t*) and *Z_j_*(*t*).

Step 2.2: Collect the local minima of *Z_r_*(*t*) and Z*_j_*(*t*) by cubic spline lines to generate the lower envelopes of *Z_r_*(*t*) and *Z_j_*(*t*).

Step 2.3: The mean values of upper and lower envelopes of *Z_r_*(*t*) and Z*_j_*(*t*) are denoted as *med_r_*_1_ and *med_j_*_1_, respectively. In addition, the differences *h_r_*_1_ and *h_j_*_1_ between the upper and lower envelopes of *Z_r_*(*t*) and *Z_j_*(*t*) and their mean values are obtained using Equation (5). Ideally, *h_r_*_1_ and *h_j_*_1_ should be the first IMF components of *Z_r_*(*t*) and *Z_j_*(*t*), respectively.

Step 2.4: If *h_r_*_1_ and *h_j_*_1_ do not satisfy the IMF requirements in Equations (6) and (7), *h_r_*_1_ and *h_j_*_1_ are set as the original signals and repeat steps 2.1–2.3 until the requirements in Equations (6) and (7) are satisfied. The obtained first IMF components of *Z_r_*(t) and *Z_j_*(*t*) are denoted as *c_r_*_1_ and *c_j_*_1_, respectively. Then, obtain the residues *r_r_*_1_ and *r_j_*_1_ by subtracting *c_r_*_1_ and c*_j_*_1_ from their original signals in Equation (8), respectively.

Step 2.5: If *r_r_*_1_ and *r_j_*_1_ still contain dynamic information of other components, treat them as new data and repeat the above sifting process to obtain the next IMFs *c_r_*_2_ and *c_j_*_2_. Such a process is repeated until the predetermined criteria shown in Equation (9) reach values in the range of 0.2~0.3.

Step 2.6: If the sifting process satisfies the IMF requirements in Equations (6) and (7), *Z_r_*(*t*) and *Z_j_*(*t*) are separately decomposed into *n* IMFs, and *c_r_*_1_-*c_rn_*, *c_j_*_1_-*c_jn_*, and the final residues *r_rn_* and *r_jn_* are obtained using Equation (10).

Step 3: The decomposed IMFs are obtained using Equation (11).

Step 4: Construct the wavelet dictionary Ψ using Equation (16); then, compute the correlation coefficient $K_{r}^{i}$ in Equations (18) and (19):

(18) $$\langle \psi_{r}\left(t\right),imf_{i}\rangle =||\psi_{r}||{}_{2}||imf_{i}||{}_{2}\cos\theta ,$$

(19) $$K_{r}^{i}=\cos\theta =\sqrt{2}\frac{\left|\langle \psi_{r}\left(t\right),imf_{i}\rangle \right|}{||\psi_{r}||{}_{2}||imf_{i}||{}_{2}},$$

where *imf_i_* is the *i*th mode of the original vibration signal, *ψ_r_*(*t*) is one of the dictionaries of wavelets Ψ, and $K_{r}^{i}$ is a matrix whose dimensions are determined by the parameter vector {*ω*, *ζ*, *τ*}. Define $\bar{\omega }$*_i_* and $\bar{\zeta }$*_i_* as parameters of the Laplace wavelet associated with the peak correlation. The relationship between the peak value generated in this progress $K_{\tau }^{i}$ and a local peak value $K_{r}^{\tau i}$ is

(20) $$K_{\tau }^{i}=\underset{\begin{array}{c} \omega \in \Omega \\ \zeta \epsilon Z \end{array}}{\max}K_{r}^{\tau i}=K_{\left\{\bar{\omega },\bar{\zeta ,}\tau_{i}\right\}}.$$

The given frequency $\bar{\omega }$*_i_* and damping factor $\bar{\zeta }$*_i_* associated with the peak values $K_{\tau }^{i}$ can represent the modal information of the *i*th mode of a wooden utility pole.

## 4. Non-Destructive Inspection of Wooden Utility Poles in the Field

Field testing was conducted on three wooden utility poles. The embedment conditions of the three tested wooden utility poles were similar, which were firm clay. The codes of these poles were Nos. 57704, 244984, and 265173. A measurement system in this study contained a PCB impact hammer, four PCB accelerometers, and a four-channel Spectral Dynamics Bobcat spectrum analyzer.

### 4.1. Testing Setup

The PCB impact hammer was used in field testing to excite vibrations of wooden utility poles. Impact hammer excitation is in a valid range that can make the power spectral density of a vibration signal at frequencies up to 800 Hz. Since the PCB impact hammer has a wide effective frequency range of up to 800 Hz, more vibration information of wooden utility poles can be extracted from their frequency response functions. The configuration parameters of the impact hammer are shown in Table 1. Four PCB accelerometers were used in field testing for data collection. The parameters of the PCB accelerometers are listed in Table 2. The Spectral Dynamics Bobcat spectrum analyzer was used in field testing for data acquisition and analysis. Specifications of the Bobcat spectrum analyzer are listed in Table 3. During the wooden utility pole testing, the environment temperature remains within 17 °C to 20 °C. The moisture contents of the tested wooden utility poles in the third inch from the ground were in the range from 25% to 30% [26]. The influences of the environmental temperature, moisture contents of wooden utility poles, and impact hammer excitation on vibration testing results were small.

**Figure 4:**
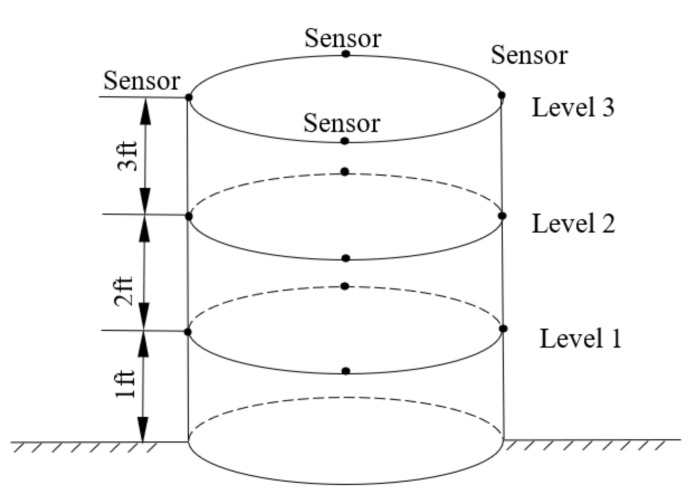
Measurement point assignments of each wooden utility pole.

Some detailed information about the three tested wooden utility poles is listed in Table 4. The measurement location of each accelerometer or sensor is shown in Figure 4. The four accelerometers were placed at 90° from each other on the same layer circle, and there were three levels for each tested wooden utility pole. Due to the symmetry of the three wooden utility poles, the data collected from each accelerometer at every level had similar information. The data from sensor 4 at level 1 of each tested wooden utility pole was analyzed in field testing here.

### 4.2. Extraction of Instantaneous Frequencies and Damping Factors of Decomposed Signals of Dynamic Responses of Wooden Utility Poles

To obtain the relationship between instantaneous frequencies and damping factors of decomposed signals of dynamic responses of tested wooden utility poles and their health conditions, the instantaneous frequency and damping factor of the decomposed signal of each single mode needed to be extracted from multiple closely spaced modes of the acquired vibration signal with high precision.

**Figure 5:**
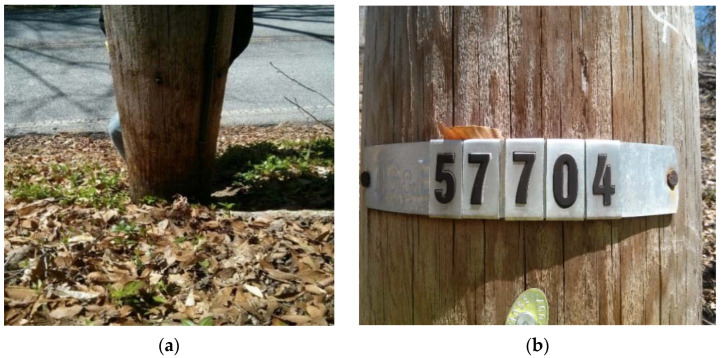
Tested wooden utility pole labeled as 57704: (**a**) its embedment condition and (**b**) its label.

**Figure 6:**
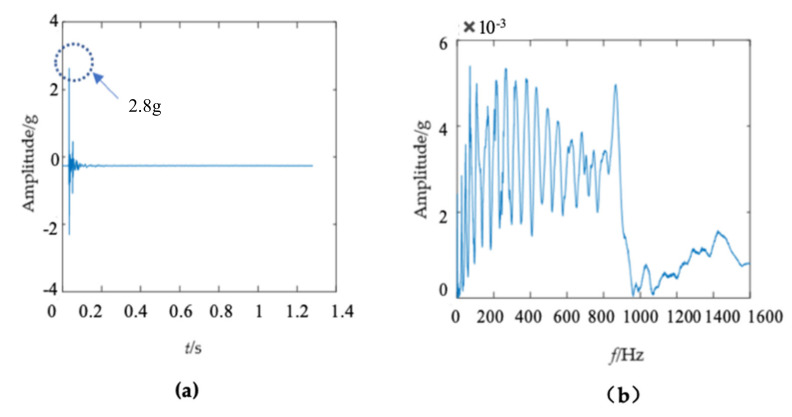
(**a**) Vibration response signal of the wooden utility pole labeled as 57704 in the time domain and (**b**) its corresponding frequency spectrum.

A modal test was conducted on a wooden utility pole labeled as 57704, as shown in Figure 5. The acquired vibration signal is shown in Figure 6. The impact response signal in Figure 6a quickly damped out in about 0.1 s. The maximum amplitude of the waveform was about 3 g in the time domain. There were many confusing closely spaced peaks in the frequency domain in Figure 6b due to nonlinear properties of the wooden utility pole, where spectral analysis of acquired vibration signals was conducted using MATLAB via the fast Fourier transform. In order to identify changes in the instantaneous frequency and damping factor of the decomposed signal of each single mode in different health conditions of a wooden utility pole, it was required to decompose multiple close modes into several single modes.

**Figure 7:**
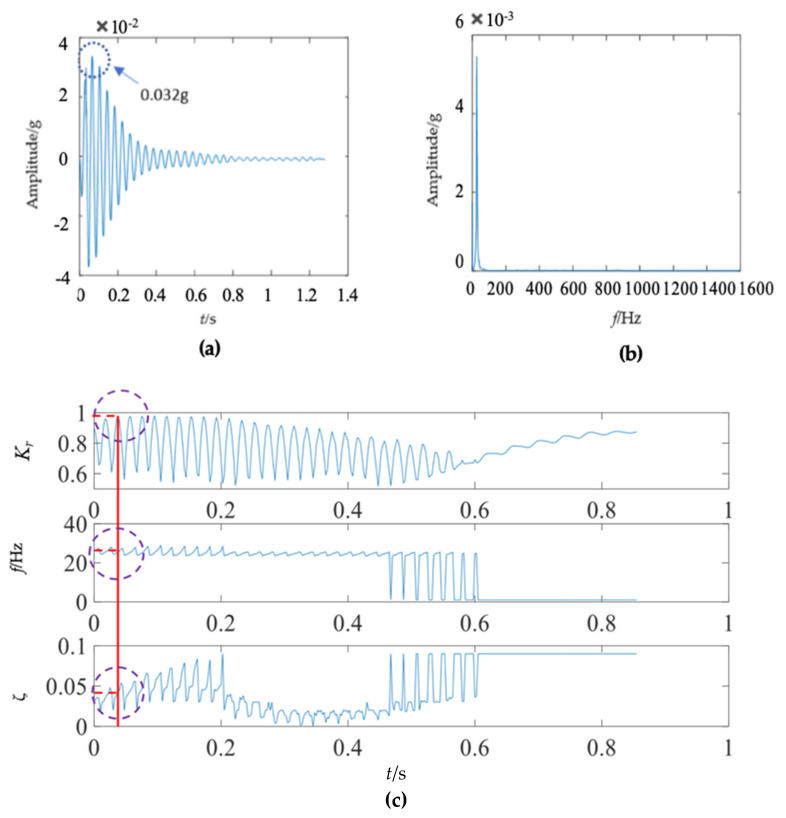
First mode in the vibration response signal of the wooden utility pole labeled as 57704: (**a**) the vibration response of the first mode in the time domain, (**b**) the frequency spectrum of the first mode, and (**c**) the results of Laplace wavelet correlation filtering of the first mode.

**Figure 8:**
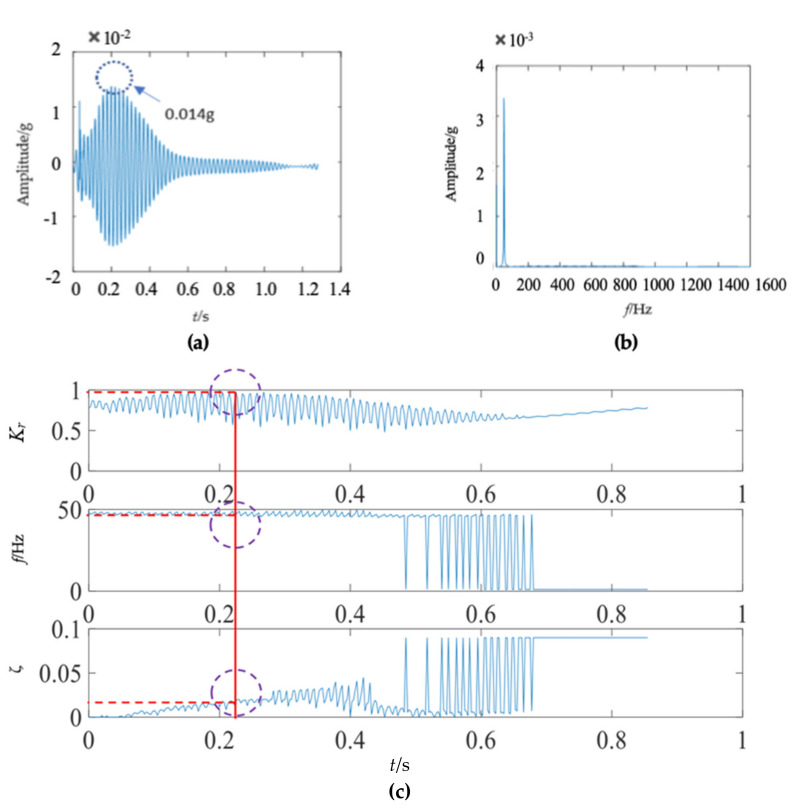
Second mode in the vibration response signal of the wooden utility pole labeled as 57704: (**a**) the vibration response of the second mode in the time domain, (**b**) the frequency spectrum of the second mode, and (**c**) the results of Laplace wavelet correlation filtering of the second mode.

**Figure 9:**
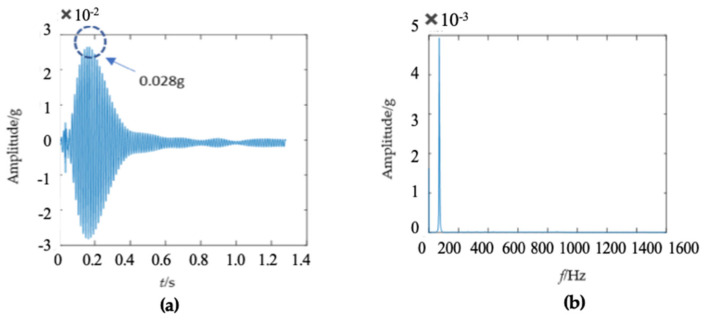

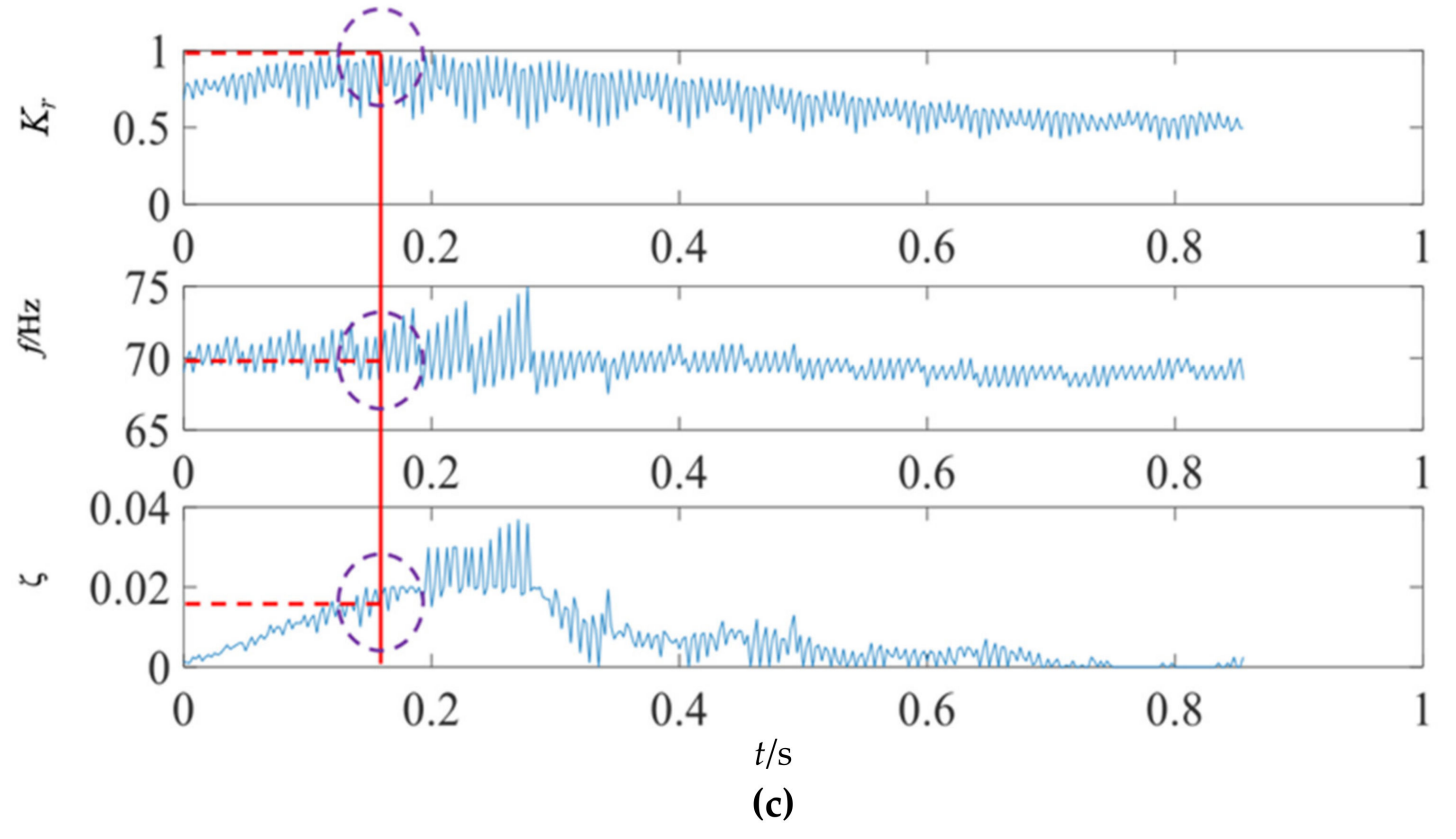
Third mode in the vibration response signal of the wooden utility pole labeled as 57704: (**a**) the vibration response of the third mode in the time domain, (**b**) the frequency spectrum of the third mode, and (**c**) the results of Laplace wavelet correlation filtering of the third mode.

The first mode of the vibration response signal of the wooden utility pole is shown in Figure 7. The instantaneous frequency and damping factor of the decomposed signal of each single mode were extracted using Laplace wavelet correlation. The result of the correlation coefficient *K_τ_* was close to 1 when a particular Laplace wavelet successfully matched with the single mode of the signal, as shown in Figure 7c. The second and third subgraphs in Figure 7c indicated the instantaneous frequency $\bar{f}$ and damping factor $\bar{\zeta }$ with the maximum correlation coefficient *K_τ_* at time *τ*. The red vertical line in Figure 7c indicated the time *τ* when the correlation coefficient *K_τ_* reached the maximum value. The purple dashed circles indicated the instantaneous frequency $\bar{f}$ and damping factor $\bar{\zeta }$ corresponding to the maximum correlation coefficient *K_τ_* at time *τ*. The decomposed vibration response of each single mode in the time domain, its corresponding frequency spectrum, and the results of Laplace wavelet correlation filtering were also obtained. Due to the space limitation of this paper, only the results of the first three modes are displayed in Figure 7, Figure 8 and Figure 9. The maximum correlation coefficients *K_τ_* of the first three modes of the wooden utility pole labeled as 57704 were 0.981, 0.9798, and 0.9841, as shown in Figure 7c, Figure 8c and Figure 9c, respectively. Instantaneous frequencies of the first three modes of the wooden utility pole labeled as 57704 were 25.5 Hz at 0.0375 s, 47 Hz at 0.225 s, and 70 Hz at 0.16 s, respectively. The damping factors $\bar{\zeta }$ with the maximum correlation coefficients *K_τ_* of the first three modes were 0.042, 0.0185, and 0.018, respectively. Instantaneous frequencies and damping factors of decomposed signals of the first 20 modes are listed in Table 5.

The instantaneous frequency and damping factor of the decomposed signal of each single mode and the correlation coefficient *K_τ_* between each single mode and constructed Laplace wavelet atoms were obtained using the proposed method, as shown in Table 5. Most correlation coefficients *K_τ_* in Table 5 were larger than 0.95, which was close to 1, which meant that the matching degree between each single mode and the Laplace wavelet was excellent. Therefore, the extracted instantaneous frequency and damping factor of the decomposed signal of each single mode could be used to reflect the vibration response information of the wooden utility pole.

**Figure 10:**
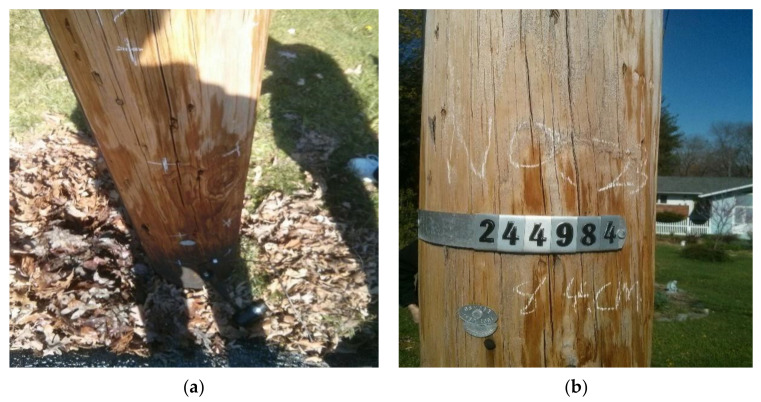
Tested wooden utility pole labeled as 244984: (**a**) its embedment condition and (**b**) its label.

**Figure 11:**
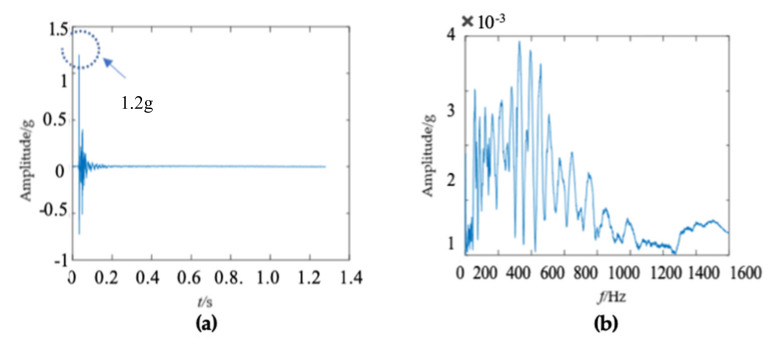
Vibration response signal and frequency spectrum of the wooden utility pole labeled as 244984: (**a**) the vibration response in the time domain and (**b**) the frequency spectrum.

The second wooden utility pole labeled as 244984, as shown in Figure 10, was also tested. The acquired waveform in the time domain is shown in Figure 11a and the impact response signal quickly damped out in about 0.2 s; the time duration of the second wooden utility pole was a little longer than that of the first wooden utility pole shown in Figure 6a. The maximum amplitude of the waveform was about 1.2 g in the time domain, which was also smaller than that of the first wooden utility pole in Figure 6a.

**Figure 12:**
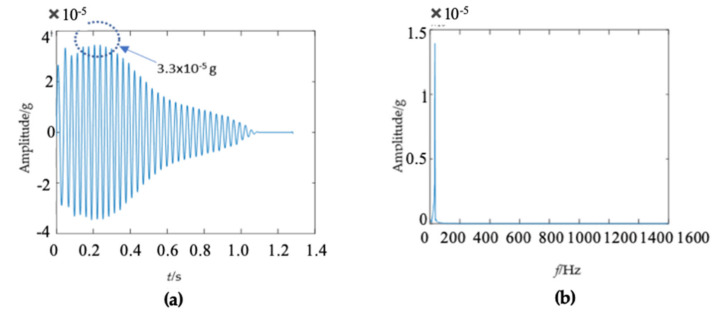

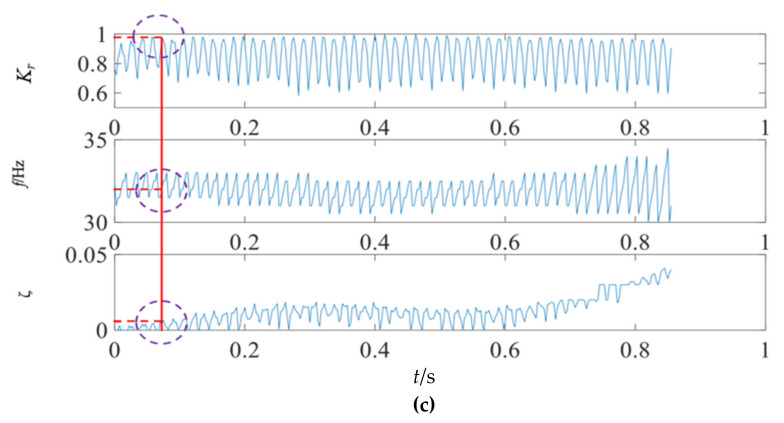
First mode in the vibration response signal of the wooden utility pole labeled as 244984: (**a**) the vibration response of the first mode in the time domain, (**b**) the frequency spectrum of the first mode, and (**c**) the results of Laplace wavelet correlation filtering of the first mode.

**Figure 13:**
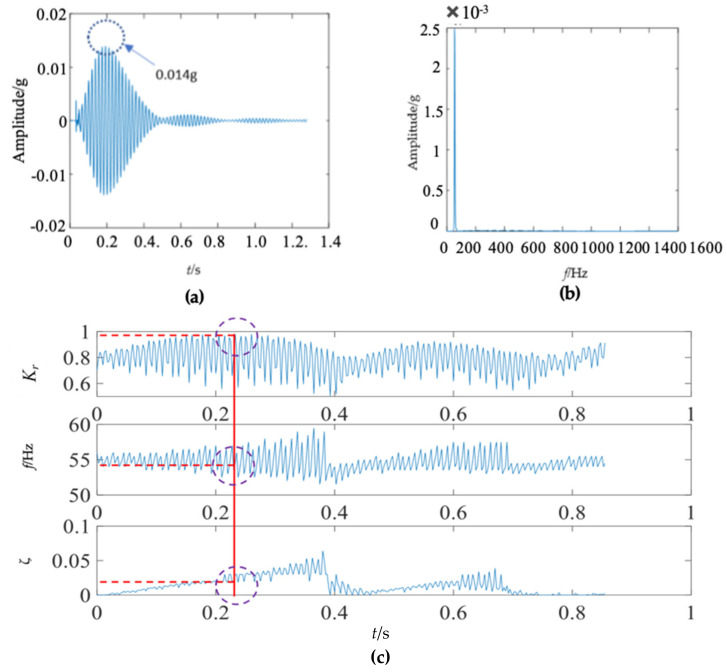
Second mode in the vibration response signal of the wooden utility pole labeled as 244984: (**a**) the vibration response of the second mode in the time domain, (**b**) the frequency spectrum of the second mode, and (**c**) the results of Laplace wavelet correlation filtering of the second mode.

**Figure 14:**
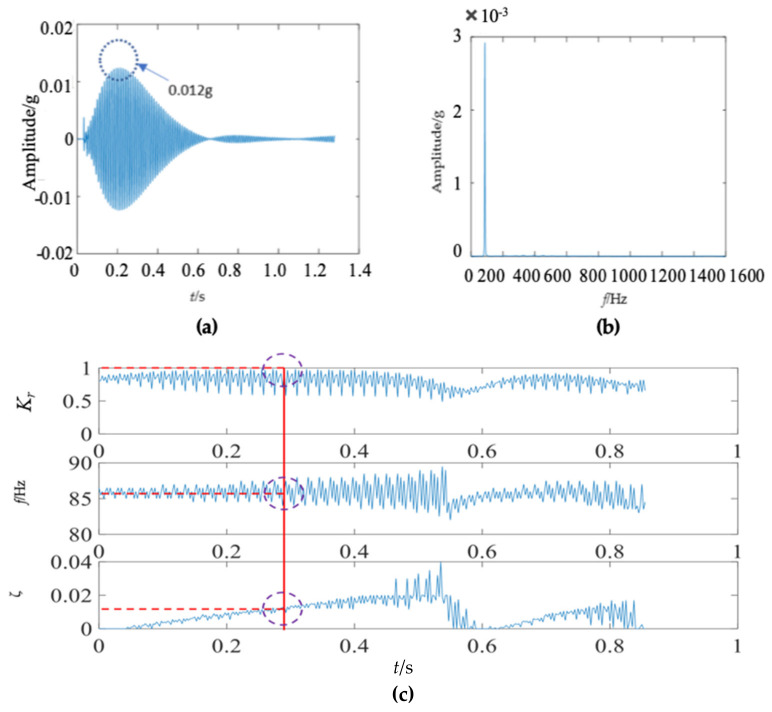
Third mode in the vibration response signal of the wooden pole labeled as 244984: (**a**) the vibration response of the third mode in the time domain, (**b**) the frequency spectrum of the third mode, and (**c**) the results of Laplace wavelet correlation filtering of the third mode.

There were some differences between the frequency spectra of the first and second wooden utility poles, as shown in Figure 6b and Figure 11b, respectively; the shapes and distributions of their spectra were different from each other. The spectrum of the first wooden utility pole in Figure 6b was mainly distributed in two clearly distinguishable regions. Most spectral lines were located in the low- and medium-frequency regions between 0 Hz and 1000 Hz, and they had dominating vibration energies since the amplitudes of the frequency components in this frequency region were relatively larger than those in the high-frequency region between 1000 Hz and 1600 Hz. Although most spectral lines were also located in the low- and medium-frequency region between 0 Hz and 1000 Hz in Figure 11b, the difference between the frequency spectra in the low/medium- and high-frequency regions was not as obvious as that in Figure 6b. The vibration signal of the second wooden utility pole was decomposed into the first 20 separated single modes using the proposed FM-EMD method and Laplace wavelet correlation filtering. The vibration responses in the time domain and the frequency spectra and Laplace wavelet correlation filtering of the first three single modes are shown in Figure 12, Figure 13 and Figure 14. Maximum correlation coefficients *K_τ_* of the first three modes of the wooden utility pole labeled as 244984 were 0.9823, 0.9813, and 0.9858, as shown in Figure 12c, Figure 13c and Figure 14c, respectively. Instantaneous frequencies of the first three modes of the wooden utility pole labeled as 244984 were 32 Hz at 0.0575 s, 54.5 Hz at 0.2325 s, and 86 Hz at 0.2725 s, respectively. The damping factors $\bar{\zeta }$ with the maximum correlation coefficients *K_τ_* of the first three modes were 0.004, 0.03, and 0.0125, respectively. The instantaneous frequencies and damping factors of decomposed signals of the first 20 modes of the wooden utility pole labeled as 244984 were extracted and are listed in Table 6.

**Figure 15:**
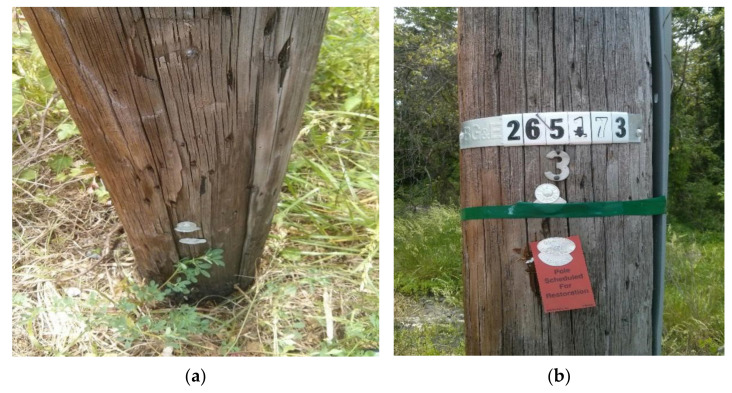
Tested wooden utility pole labeled as 265173: (**a**) its embedment condition and (**b**) its label.

**Figure 16:**
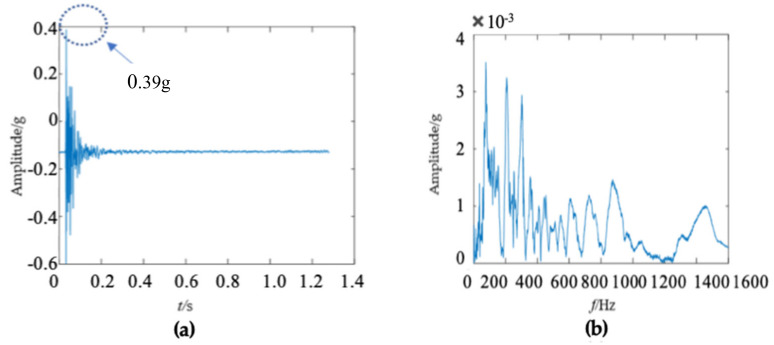
Vibration response signal and frequency spectrum of the wooden utility pole labeled as 265173: (**a**) the vibration response in the time domain and (**b**) the frequency spectrum.

The third wooden utility pole labeled as 265173, as shown in Figure 15, was also tested. The testing process was the same as those for the above two wooden utility poles labeled as 57704 and 244984. The acquired waveform in the time domain is shown in Figure 16a. It was found that the impact response signal damped out in 0.4 *s*, which was longer than those of the above two wooden utility poles in Figure 6a and Figure 11a. The maximum amplitude of the waveform was about 0.4g in the time domain, which was also smaller than those of the above two wooden utility poles.

**Figure 17:**
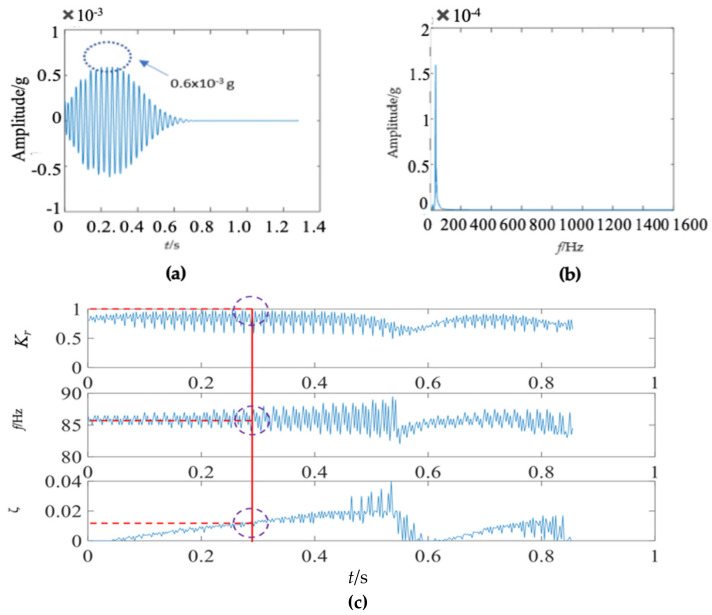
First mode in the vibration response signal of the wooden utility pole labeled as 265173: (**a**) the vibration response of the first mode in the time domain, (**b**) the frequency spectrum of the first mode, and (**c**) the results of Laplace wavelet correlation filtering of the first mode.

**Figure 18:**
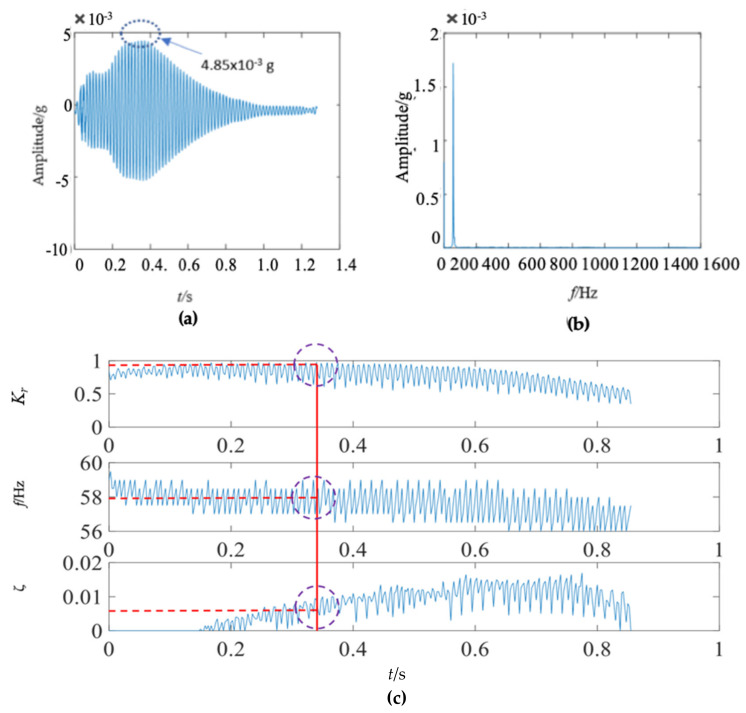
Second mode in the vibration response signal of the wooden utility pole labeled as 265173: (**a**) the vibration response of the second mode in the time domain, (**b**) the frequency spectrum of the second mode, (**c**) and the results of Laplace wavelet correlation filtering of the second mode.

**Figure 19:**
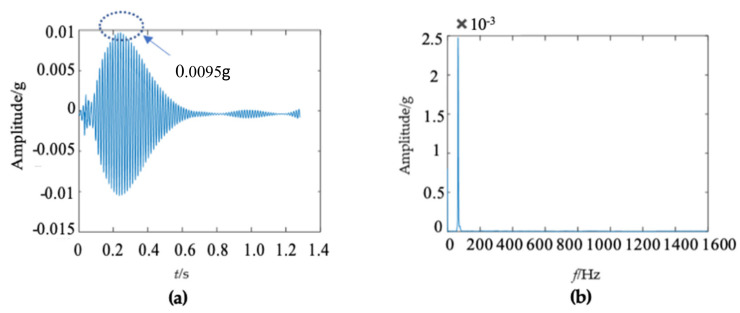

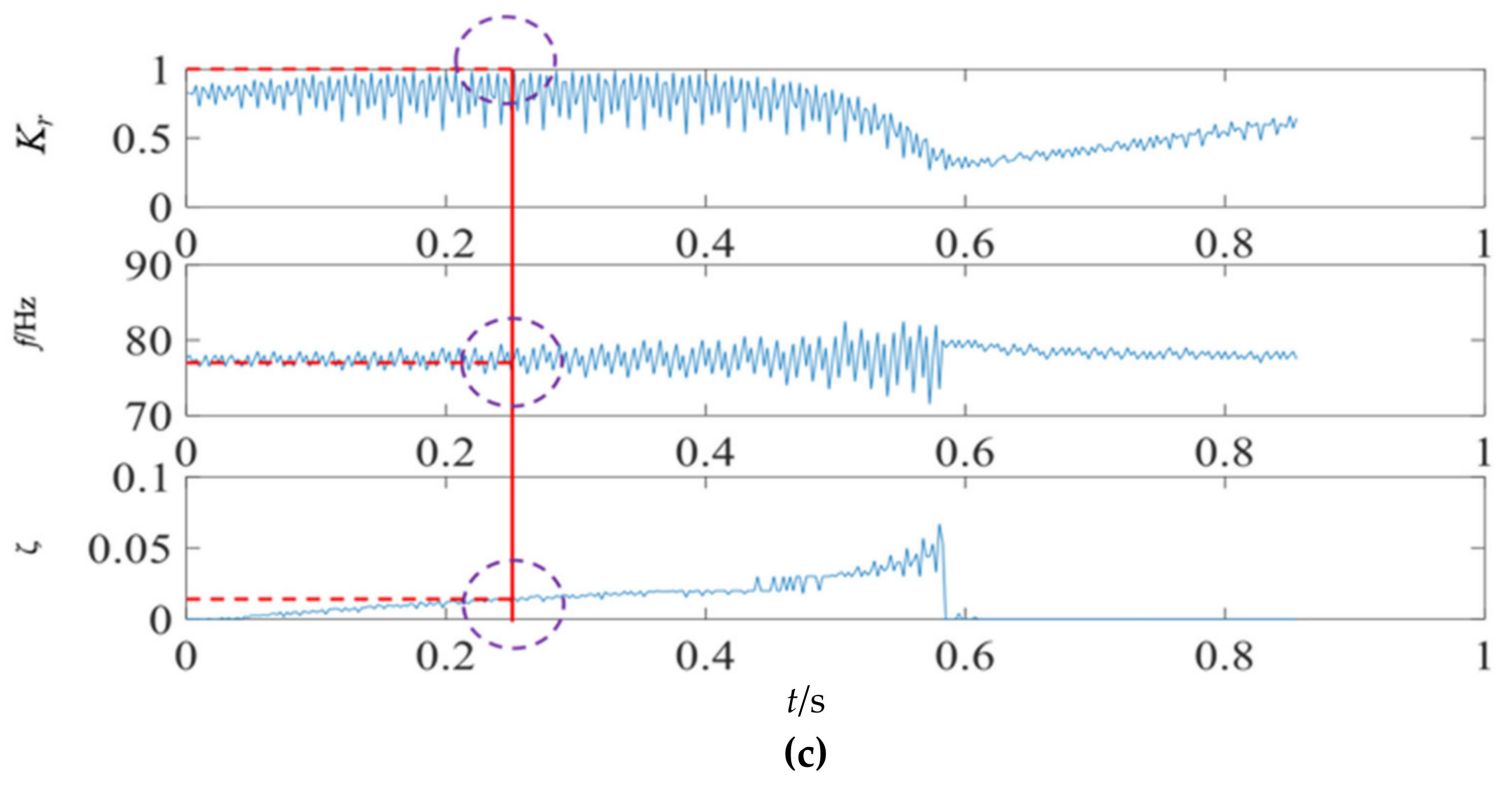
Third mode in the vibration response signal of the wooden utility pole labeled as 265173: (**a**) the vibration response of the third mode in the time domain, (**b**) the frequency spectrum of the third mode, (**c**) and the results of Laplace wavelet correlation filtering of the third mode.

There were some differences in the frequency spectra of the three wooden utility poles in Figure 6b, Figure 11b and Figure 16b. The shape and distribution of the spectrum in Figure 16b were different from those of the other two wooden utility poles in Figure 6b and Figure 11b. Larger spectral peaks mainly appeared in the low- and medium-frequency regions and peak amplitudes in the high-frequency region were closer to those in the low- and medium-frequency regions. Therefore, the energy of the spectrum increased with the frequency in the low/medium- and high-frequency regions and it was more uniformly distributed in Figure 16b. Moreover, the maximum amplitude of the frequency spectrum of the wooden utility pole labeled as 265173 was the smallest among the three wooden utility poles. The acquired vibration signal was decomposed into 20 separated single modes using the proposed FM-EMD method and Laplace wavelet correlation filtering. Similar to the first two wooden utility poles labeled as 57704 and 244984, vibration responses in the time domain, their frequency spectra, and the results of Laplace wavelet correlation filtering of the first three modes are shown in Figure 17, Figure 18 and Figure 19. The maximum correlation coefficients *K_τ_* of the first three modes of the wooden utility pole labeled as 265173 were 0.8847, 0.9768, 0.9668, as shown in Figure 17c, Figure 18c and Figure 19c, respectively. The instantaneous frequencies of the first three modes of the wooden utility pole labeled as 265173 were 37 Hz at 0.03 s, 58 Hz at 0.34 s, and 65.5 Hz at 0.155 s, respectively. The damping factors $\bar{\zeta }$ with the maximum correlation coefficients *K_τ_* of the first three modes were 0.019, 0.009, and 0.014, respectively. The instantaneous frequencies and damping factors of decomposed signals of the first 20 modes of the wooden utility pole labeled as 265173 are listed in Table 7.

### 4.3. Health Evaluation of the Three Wooden Utility Poles Based on Instantaneous Frequencies and Damping Factors

**Figure 20:**
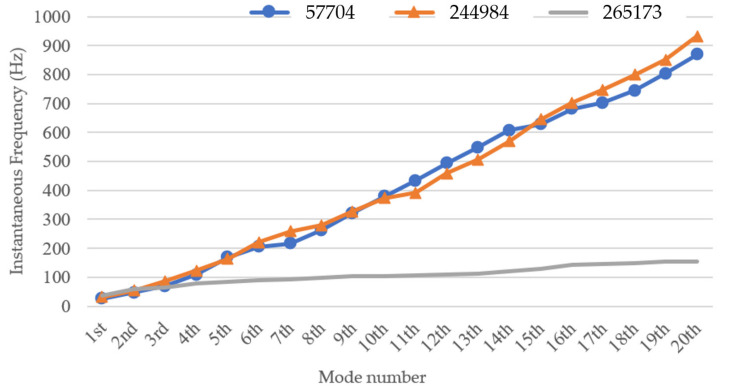
Instantaneous frequencies of decomposed signals of the first 20 modes of the three wooden utility poles.

**Figure 21:**
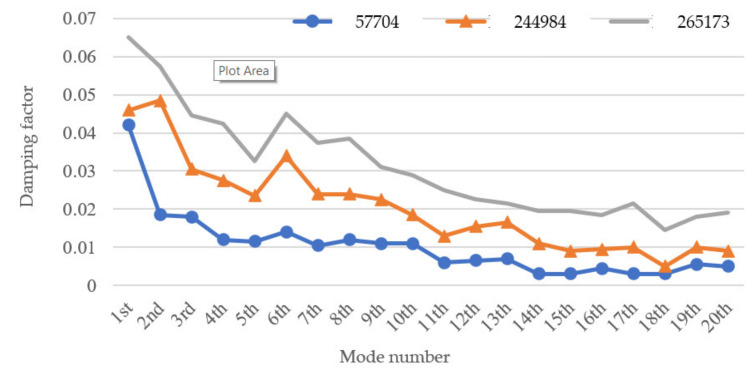
Damping factors of decomposed signals of the first 20 modes of the three wooden utility poles.

In order to observe the rules of the obtained instantaneous frequencies and damping factors from measured vibration response signals of the three wooden utility poles labeled as 57704, 244984, and 265173, the extracted instantaneous frequencies and damping factors of the three wooden utility poles are shown in Figure 20 and Figure 21, respectively.

It can be seen from Figure 20 that the instantaneous frequencies varied approximately linearly from the 1st to the 20th modes of the three wooden utility poles. The instantaneous frequencies of the wooden utility pole labeled as 265173 were much smaller than those of the wooden utility poles labeled as 57704 and 244984, which could mean that the stiffness of the wooden utility pole labeled as 265173 was smaller than those of the other two wooden utility poles, and there might exist some damage in this wooden utility pole. In addition, the instantaneous frequencies of decomposed signals of the wooden utility pole labeled as 244984 were similar to those of the wooden utility pole labeled as 57704. Therefore, there was a preliminary diagnosis conclusion that wooden utility poles labeled 57704 and 244984 could be healthy and the wooden utility pole labeled as 265173 could be damaged.

According to the damping factor results of the three wooden utility poles in Figure 21, the damping factors of the damaged wooden utility pole labeled as 265173 were larger than those of the two healthy wooden utility poles. Larger damping factors indicated that there could be damage in the wooden utility poles. Noticeably, all the damping factors of the wooden utility pole labeled as 244984 were larger than those of the wooden utility pole labeled as 57704, which indicated that the wooden utility labeled as 57704 was healthier than the wooden utility pole labeled as 244984. It is noted that the use of damping factors can more clearly distinguish the health conditions between the wooden utility poles labeled as 244984 and 57704 than the use of instantaneous frequencies. 

The traditional hole-drilling inspection test was applied to the three wooden utility poles to validate the results from the proposed method. The traditional test results showed that there was decay inside the wooden utility pole labeled as 265173; the pole was indeed considered damaged and it should be immediately repaired or replaced. The traditional test results showed that the other two poles were healthy. While there might exist some minor damage in the wooden utility pole labeled as 244984, the damage did not affect its usual usage and the pole was judged as healthy. However, it needed constant attention and maintenance. The traditional test results were consistent with the results from the proposed method.

## 5. Conclusions

The instantaneous frequency and damping factor of the decomposed signal of each single mode of the measured vibration response signal of a wooden utility pole could be extracted to realize non-destructive health evaluation. The proposed FM-EMD method could decompose multiple modes of a wooden utility pole into several single modes. The instantaneous frequency and damping factor of the decomposed signal of each single mode could be identified using Laplace wavelet correlation filtering.

The instantaneous frequency and damping factor of the decomposed signal of each single mode of each of the three wooden utility poles labeled as 57704, 244984, and 265173 were successfully used to determine their health conditions. The instantaneous frequencies of the first 20 modes of the wooden utility pole labeled as 265173 changed from 37 Hz to 83.5 Hz, as shown in Figure 20. The instantaneous frequencies of the first 20 modes of wooden poles labeled as 57704 and 244984 changed from 25.5 Hz to 168 Hz and from 32 Hz to 163.5 Hz, respectively. The increase rate of instantaneous frequencies of the first 20 modes of the wooden utility pole labeled as 265173 with the mode number was much smaller than those of the wooden utility poles labeled as 57704 and 244984. Based on the increase rates of the instantaneous frequencies of the first 20 modes of the wooden utility poles, it was judged that the lower stiffness increase rate of the wooden utility pole labeled as 265173 was caused by some damage in the pole. Additionally, the damping factors of the first 20 modes of the wooden utility pole labeled as 265173 were larger than those of wooden poles labeled as 244984 and 57704. The damping factors of the wooden utility pole labeled as 244984 were noticeably larger than those of the wooden utility pole labeled as 57705 since the latter was healthier than the former. Larger damping factors of the wooden utility pole labeled as 265173 indicated that it could be damaged. Consistent with the traditional hole-drilling inspection test results, the wooden utility poles labeled as 57704 and 244984 were deemed healthy, and the wooden utility pole labeled as 265173 was damaged. Some maintenance suggestions for the two wooden utility poles labeled as 265173 and 244984 can be drawn: the wooden utility pole labeled as 265173 must be immediately repaired or replaced to avoid a potential failure accident and the wooden utility pole labeled as 244984 needed some maintenance to strengthen it.

## Figures and Tables

**Table 1:** Configuration parameters of the PCB impact hammer.

Item	Value
Product model	PCB 086D20
Sensitivity	1 mV/N
Measurement range	±5000 N
Resonant frequency	≥12 kHz
Constant current excitation	2 to 20 mA
Excitation voltage	20 to 30 V
Hammer mass	2.4 lb

**Table 2:** Detailed parameters of the PCB accelerometers.

Item	Value
Product model	PCB 352C66
Sensitivity	100 mV/g
Measurement range	±50 g
Resonant frequency	≥35 kHz
Frequency range	0.5 to 10,000 Hz
Phase response	2 to 6000 Hz
Broadband resolution	0.00016g rms

Note: g is the acceleration of gravity and rms means root mean square.

**Table 3:** Specifications of the Bobcat spectrum analyzer.

Item	Value
Product model	Spectral Dynamics Bobcat Shaker Controller
Number of input channels	2
Number of output channels	4
Voltage range	27 mV to 10 V
Maximum output amplitude	±12 V
Maximum output current	16 mA
Sampling rate	51,200 samples per second

**Table 4:** Detailed descriptions and parameters of the tested wooden utility poles.

Item	Description or Value
Wood species	Southern yellow pine
Wood type	Hardwood
National standard class	2
Cross-section shape	Circular
Pole length	9144 mm
Groundline distance from the button	1676 mm
Circumference 6 feet from the button	865 mm
Circumference at the top	635 mm

**Table 5:** Instantaneous frequencies and damping factors of decomposed signals of the first 20 modes of the wooden utility pole labeled as 57704.

Mode No.	Max(*K*_τ_)	Corresponding Time (s)	Instantaneous Frequency (*f/*Hz)	Damping Factor (ζ)
1st	0.981	0.038	25.50	0.042
2nd	0.980	0.225	47.00	0.019
3rd	0.984	0.160	70.00	0.018
4th	0.984	0.205	108.0	0.012
5th	0.969	0.165	168.0	0.012
6th	0.969	0.088	204.5	0.014
7th	0.951	0.143	216.0	0.011
8th	0.966	0.070	262.0	0.012
9th	0.963	0.105	322.0	0.011
10th	0.960	0.103	378.0	0.011
11th	0.962	0.075	434.5	0.006
12th	0.962	0.075	493.0	0.007
13th	0.954	0.090	549.5	0.007
14th	0.901	0.045	606.5	0.003
15th	0.947	0.088	628.0	0.003
16th	0.959	0.070	682.0	0.005
17th	0.940	0.073	703.5	0.003
18th	0.949	0.075	745.5	0.003
19th	0.954	0.078	803.0	0.006
20th	0.946	0.068	869.5	0.005

**Table 6:** Instantaneous frequencies and damping factors of decomposed signals of the first 20 modes of the wooden utility pole labeled as 244984.

Mode No.	Max(*K_τ_*)	Corresponding Time (s)	Instantaneous Frequency (*f/*Hz)	Damping Factor (ζ)
1st	0.982	0.058	32.00	0.004
2nd	0.981	0.233	54.50	0.030
3rd	0.986	0.273	86.00	0.013
4th	0.983	0.173	122.0	0.016
5th	0.986	0.190	163.5	0.012
6th	0.981	0.098	220.0	0.020
7th	0.982	0.135	257.0	0.014
8th	0.979	0.163	279.5	0.012
9th	0.984	0.114	326.5	0.012
10th	0.977	0.110	372.0	0.008
11th	0.984	0.113	392.0	0.007
12th	0.984	0.125	458.0	0.009
13th	0.985	0.093	506.0	0.010
14th	0.981	0.118	570.0	0.008
15th	0.983	0.088	645.5	0.006
16th	0.982	0.150	702.0	0.005
17th	0.989	0.098	747.0	0.007
18th	0.936	0.130	799.0	0.002
19th	0.987	0.110	852.5	0.005
20th	0.960	0.125	931.5	0.004

**Table 7:** Instantaneous frequencies and damping factors of decomposed signals of the first 20 modes of the wooden utility pole labeled as 265173.

Mode No.	Max(*K_τ_*)	Corresponding Time (*s*)	Instantaneous Frequency (*f*/Hz)	Damping Factor (ζ)
1st	0.885	0.030	37.00	0.019
2nd	0.977	0.340	58.00	0.009
3rd	0.967	0.155	65.50	0.014
4th	0.993	0.253	77.50	0.015
5th	0.982	0.190	83.50	0.009
6th	0.918	0.148	89.50	0.011
7th	0.956	0.128	93.00	0.014
8th	0.920	0.083	98.00	0.015
9th	0.917	0.160	103.0	0.009
10th	0.906	0.145	104.5	0.011
11th	0.900	0.168	106.0	0.012
12th	0.941	0.193	109.5	0.007
13th	0.920	0.208	113.0	0.005
14th	0.913	0.143	119.5	0.009
15th	0.942	0.130	128.5	0.011
16th	0.911	0.100	144.0	0.009
17th	0.916	0.093	146.5	0.012
18th	0.904	0.085	148.0	0.010
19th	0.932	0.120	153.5	0.008
20th	0.940	0.125	155.5	0.010
